# RNA encoded peptide barcodes enable efficient *in vivo* screening of RNA delivery systems

**DOI:** 10.1093/nar/gkae648

**Published:** 2024-07-25

**Authors:** Uchechukwu Odunze, Nitin Rustogi, Paul Devine, Lorraine Miller, Sara Pereira, Surender Vashist, Harm Jan Snijder, Dominic Corkill, Alan Sabirsh, Julie Douthwaite, Nick Bond, Arpan Desai

**Affiliations:** Cell, Gene and RNA Therapy, Discovery Sciences, R&D, AstraZeneca, Cambridge, UK; Advanced Drug Delivery, Pharmaceutical Sciences, R&D, AstraZeneca, Cambridge, UK; Physico-chemical Development, Biopharmaceuticals Development, R&D, AstraZeneca, Cambridge, UK; Physico-chemical Development, Biopharmaceuticals Development, R&D, AstraZeneca, Cambridge, UK; Animal Sciences and Technologies, Clinical Pharmacology and Safety Sciences, R&D, AstraZeneca, Cambridge, UK; Advanced Drug Delivery, Pharmaceutical Sciences, R&D, AstraZeneca, Cambridge, UK; Cell, Gene and RNA Therapy, Discovery Sciences, R&D, AstraZeneca, Cambridge, UK; Discovery Biology, Discovery Sciences, R&D, AstraZeneca, Gothenburg, Sweden; Bioscience in vivo, Early R&I, R&D, AstraZeneca, Cambridge, UK; Advanced Drug Delivery, Pharmaceutical Sciences, R&D, AstraZeneca, Gothenburg, Sweden; Cell, Gene and RNA Therapy, Discovery Sciences, R&D, AstraZeneca, Cambridge, UK; Physico-chemical Development, Biopharmaceuticals Development, R&D, AstraZeneca, Cambridge, UK; Advanced Drug Delivery, Pharmaceutical Sciences, R&D, AstraZeneca, Cambridge, UK

## Abstract

Lipid nanoparticles (LNPs) have been demonstrated to hold great promise for the clinical advancement of RNA therapeutics. Continued exploration of LNPs for application in new disease areas requires identification and optimization of leads in a high throughput way. Currently available high throughput *in vivo* screening platforms are well suited to screen for cellular uptake but less so for functional cargo delivery. We report on a platform which measures functional delivery of LNPs using unique peptide ‘barcodes’. We describe the design and selection of the peptide barcodes and the evaluation of these for the screening of LNPs. We show that proteomic analysis of peptide barcodes correlates with quantification and efficacy of barcoded reporter proteins both *in vitro* and *in vivo* and, that the ranking of selected LNPs using peptide barcodes in a pool correlates with ranking using alternative methods in groups of animals treated with individual LNPs. We show that this system is sensitive, selective, and capable of reducing the size of an *in vivo* study by screening up to 10 unique formulations in a single pool, thus accelerating the discovery of new technologies for mRNA delivery.

## Graphical Abstract

**Graphical Abstract:**
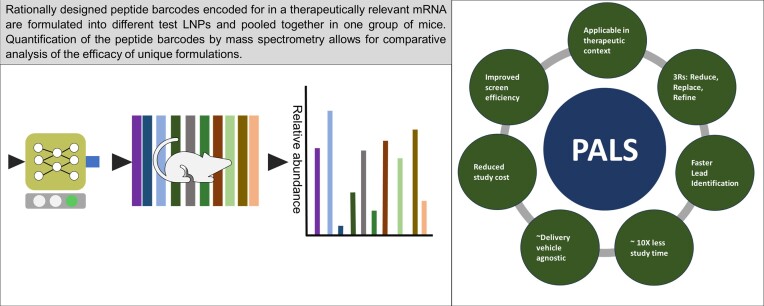

## Introduction

Lipid nanoparticles (LNPs) are enabling the rapid advancement of nucleic acid therapeutics to the clinic, including promising new modalities like CRISPR (1,2) to treat and possibly cure previously intractable diseases. LNPs typically consist of four components including polyethylene glycol (PEG)-lipids, ionizable cationic lipids, cholesterol and phospholipids in specific molar ratios. The design space for optimizing the four components of LNPs as well as the formulation parameters is very large, potentially to a scale of 10^10^ unique LNP formulations (3). This establishes the need to screen LNPs in a high throughput way.

Typically, a high throughput screen will be done *in vitro* using a suitable model and lead candidates selected for *in vivo* studies. While these *in vitro* screens help to identify promising leads, evidence of poor correlation between *in vitro* and *in vivo* results (4,5) make the case for high throughput *in vivo* screens where the particles can be assessed in a relevant physiological and pathological context. To make *in vivo* screening of LNPs more efficient, the high-throughput approach should achieve the same outcome as an individual group screen—which is lead identification and optimization based on functional delivery of the LNP cargo. Novel methods to screen for LNPs *in vivo* have been developed including the use of DNA barcodes (bDNA) to identify LNPs that target endothelial cells and other tissues (6,7). This approach allows for the screening of >100 LNPs in a single pool but requires coformulation of the functional nucleic acid (siRNA or mRNA) with a small DNA barcode. The formulation of different types of nucleic acids has however been shown to change the structure of the LNP and potentially alter the LNP delivery, potentially changing the outcome of the screen (8,9). Another approach utilizes barcodes encoded for in the 3′ untranslated (UTR) region of the functional mRNA, thus formulating only one type of nucleic acid – an improvement over the bDNA approach (9).

Advances to these methods, such as utilizing multiomics-based approaches further demonstrate the promise of high throughput screening in the development of LNPs including the ability to study the genomic (10) and cellular (11) basis for variations in nanoparticle delivery. There are however limitations in relying on bulk phenotypic read-outs like luminescence and fluorescence to assess functional delivery of effectively different LNPs in a pool as this conceals the single molecule effect of individual LNPs. Furthermore, while the cellular quantification of separate barcodes enables efficient screening for uptake and tissue tropism, there is evidence that uptake of LNPs is not always correlated with functional delivery of the LNP or the translation of an mRNA cargo (12–14). It is therefore important to distinguish between cellular or tissue uptake and functional delivery at the individual LNP level in a pool. A recent publication (15) demonstrated the application of a barcode-based screening system which relies on mass spec quantification of translated peptide barcodes encoded in the mRNA. This system offers the advantage of being able to assess functional delivery of mRNA and does not rely on the use of a reporter transgene. Using this system, the authors were able to identify a lead compound from a screen of 384 unique LNPs using 8 mice. However, the authors used monomeric streptavidin (mSA) as the carrier protein because of the ease of enrichment in tissues, which leaves the question of whether this approach can be applied using a therapeutically relevant carrier protein.

**Figure 1.**
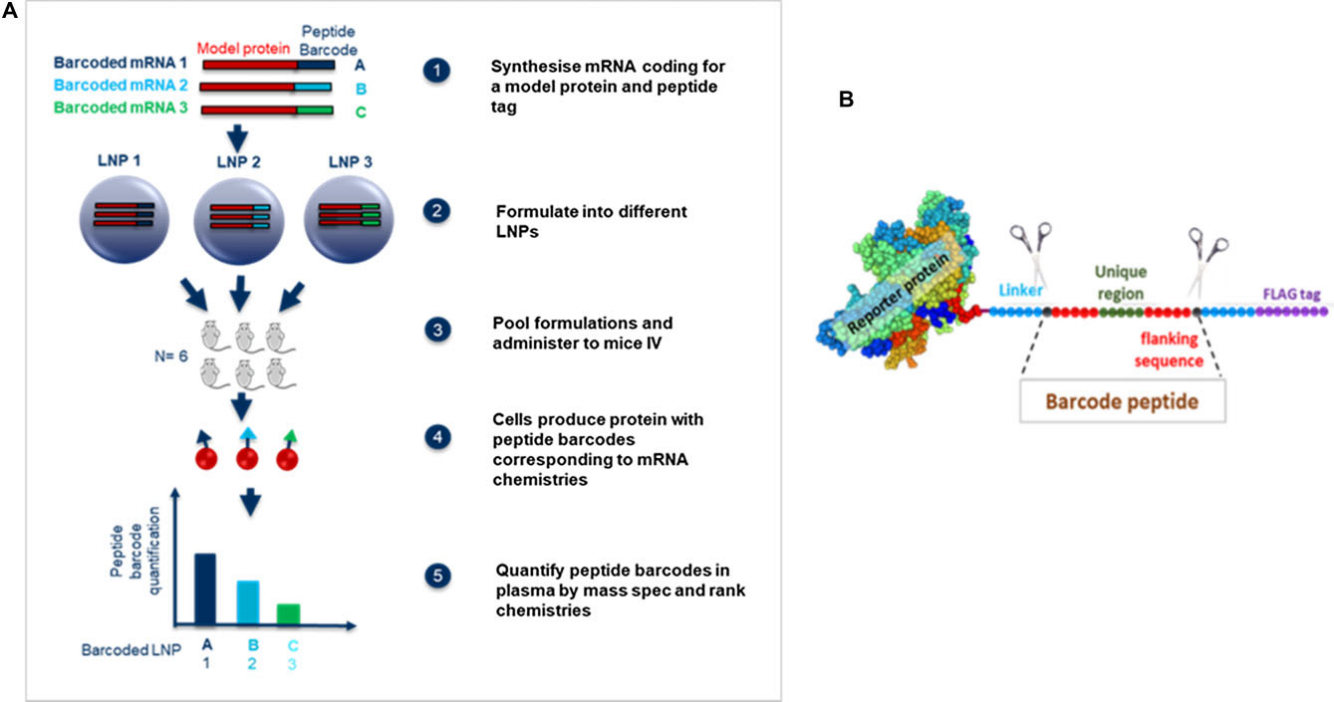
Proteomics assisted LNP screening (PALS) couples LNP uptake and functional delivery of mRNA in a single *in vivo* screen. (**A**) Illustration of experimental design. mRNA encoding a reporter/therapeutic protein is modified to contain sequence for unique peptide barcodes on the C-terminal. These barcoded mRNA are formulated into test LNPs which are pooled and administered to test animals by intravenous (i.v.) administration. The barcoded mRNA molecules are translated into functional barcoded proteins (**B**) which are collected and quantified.

We report on a similar peptide barcode-based approach and demonstrate its application in a therapeutic context, using human erythropoietin (hEPO) as the carrier protein. The Proteomics Associated LNP Screening (PALS) approach utilizes barcodes encoded for in the 3′ end of a functional mRNA molecule to screen for functional delivery of LNPs. Each barcoded mRNA (bmRNA) is formulated into a unique barcoded LNP (bLNP), pooled together, and administered to a single group of mice. The translated proteins can be collected from different tissues, enzyme digested, and the barcode peptide amount directly correlated to a unique bLNP (Figure 1A). This approach directly assesses the functionality of the LNP and the delivered cargo in a therapeutic context, thus accelerating LNP design and development for RNA therapies.

## Materials and methods

### mRNA synthesis

The sequence optimized mRNA encoding each barcoded protein- barcoded hEPO and barcoded CRE—was synthesized by *in vitro* transcription as previously described (16). Briefly, plasmid containing the optimized sequence was used to generate a linear template bearing a T7 promoter upstream of the desired sequence by PCR. The DNA template also had a 5′ untranslated region 9UTR), 3′ UTR flanking the open reading frame and a terminal polyA tail. The reaction was carried out using an IVT kit (HiScribe™ T7 High Yield RNA Synthesis Kit (E2040S), NEB) following the product guide. The reaction contained a T7 polymerase enzyme, ribonucleotide triphosphate molecules, the linear DNA template, a capping analogue, CleanCap® Reagent AG (N-7113, TriLink Biotechnologies) and modified ribonucleoside triphosphate (5-Methoxy-UTP, 5-moUTP (Trilink, N-1093) replacing uridine triphosphate (UTP). The synthesized mRNA was purified using the MEGAclear™ Transcription Clean-up kit (AM1908, ThermoFisher Scientific) and stored at –70°C. Detailed information primers, DNA sequence and PCR conditions is provided in the Supplementary Material.

**Table:** 

Thermocycling conditions for PCR (GRAD_LOW)		
Step	Temperature	Time
Initial denaturation	98°C	30 s
35 cycles	98°C	5 s
	45°C	20 s
	72°C	2 min
Final extension	72°C	10 min
Hold	4°C	

Primers:

**Table:** 

UO_temp_f	CGAATTCTAATACGACTCACTATAAGGAAATAA GAGAG
UO_temp_r	TTTTTTTTTTTTTTTTTTTTTTTTTTTTTTTTTTTTTTTTTTTTTTTTTTTTTTTTTTTTTTTTTTTTTTTTTTTTTTTTTTTTTTTTTTTTTTTTTTTTTTTTTTTTTTTTTTTTTTTcgtacGCTTCCTACTCAGGC

### Lipid nanoparticle production

LNPs were prepared using a microfluidic chip device, NanoAssemblr (Precision Nanosystems) as previously described (17). Briefly, the organic the lipid components including the ionisable lipids (Dlin-MC3-DMA), 1,2-distearoyl-*sn*-glycero-3-phosphocholine (DSPC), cholesterol (chol) and 1,2-dimyristoyl-*rac*-glycero-3-methoxypolyethylene glycol-2000 (DMG-PEG2000) were prepared as a mixture in ethanol at a ratio of 50:10:38.5:1.5. Aqueous dilutions of mRNA molecules (bmRNA, hEPO mRNA (TriLink cleancap, 5MoU) and Cre mRNA (TriLink CleanCap, 5MoU) were prepared in 50 mM citrate buffer (pH 3.0). Subsequently, the organic (lipid) and aqueous (mRNA) components were mixed in a 20:1 ratio using the NanoAssemblr in a volume ratio or 1:3 (lipids:mRNA) and flow rate of 12ml/min. LNP formulations were subsequently dialysed overnight in PBS at 4°C and then characterized.

### Cell transfection

For the *in vitro* transfection of HEP G2 cells and HEK 293 cells, lipofectamine 2000 (ThermoFisher Scientific, UK) complexed mRNA and LNP containing the desired mRNA were used. Briefly, cells were seeded into 96 well plates at a density of 5 × 10^4^ cells/well. For lipofection, mRNA was complexed with lipofectamine at a ratio of 1.5 ul lipofectamine 2000 per ug of mRNA following the manufacturer's instructions 24 h after cell seeding. 200 μl of lipoplex in Opti-MEM was added to cells and left for 24 h after which cell media was collected for subsequent analysis. For transfection using LNPs, LNP containing mRNA was diluted in Opti-MEM just before cell treatment. 100 ul of LNP suspension in Opti-MEM was added to wells already containing 100ul of 10% FBS supplemented media. Treatment was left for 24 h after which the media was collected for subsequent analysis. For fluorescent analysis, cells were imaged using an Incucyte S3 (Essen Bioscience) imager placed in an incubator. Images were captured using a 10× objective lens every 2 h for 48 h. The images were subsequently analysed using the integrated Incucyte S3 2019A software.

### Generation of recombinant barcoded EPO protein

Purified barcoded hEPO was generated by transient transfection of plasmids encoding human EPO with C-terminal barcodes followed by a FLAG tag in a HEK293 derived cell line (Patent: US 2020/0370056 A1). In brief per ml of cell culture, 15 μg plasmid DNA was mixed with 7.5 μg PEI Max incubated for 15 min prior to addition to 9-fold excess of cell culture (Expi293 Expression Medium, A14351, Life Technologies). Cells were grown in 50 ml tube spin culture tubesat 37°C with 8% CO_2_ with 250 rpm shaking. After 24 h the culture was supplemented with 10% HEK feed supplement (Xell, Cat: 871–0001) and 4 mM l-glutamine (Gibco, Cat: 25030-024). The supernatant with secreted recombinant hEPO were collected after 6 days and using a 10 kDa amicon spin concentrator. Barcoded EPO was subsequently purified using size exclusion chromatography equilibrated in phosphate buffered saline solution. Protein was concentrated on 10 kDa amicon concentrator to 1.5–2.5 mg/ml and analysed on SDS-PAGE.

### *In vivo* evaluation of LNPs

All *in vivo* procedures were carried out under the authority of a UK Project Licence which had been reviewed and approved by an Animal Welfare and Ethical Review Body (AWERB) in compliance with EU Directive 2010/63/EU before any work was carried out. Wild-type female BALB/c mice (6–8 weeks of age) were purchased from Charles River, UK and housed at the AstraZeneca animal facility. All work was carried out to Home Office UK. ethical and husbandry standards, under the authority of an appropriate project licence. A parallel group study design was followed for each study, consisting of a naïve mice control group, a positive control group of known potency and safety as well as the treatment groups. A random number generator was used to assign numbers to each mouse as they are being weighted and distributed into grouped cages containing 5 mice, based on their numbers to achieve similar group mean weights. The group size was determined using a Cohen's d power calculation which determined that a group size of 5 mice was needed to see a change equivalent to two (2) standard deviation (SD) units. A difference of two (2) standard deviations were determined to be adequate for accurate ranking of group means from a pilot study. To blind the studies, four investigators were involved in the studies: two investigators were responsible for dosing based on group allocation and sample collection post treatment, one was responsible for sample processing, and another was responsible for analysis. At the time of dosing, the mice had an average weight of 21.8 ± 0.8 g (mean ± SD). 100 ul of each LNP formulations encapsulating barcoded hEPO mRNA was administered by intravenous injection at a total mRNA dose of 0.1 mg/kg or 0.5 mg/kg. 6 h after administration, animals were terminally anaesthetized with pentobarbitone, and blood was collected. Serum was prepared and stored at –80°C for subsequent quantitation of expressed bhEPO protein. The differences between groups were assessed using One-way ANOVA with post hoc testing (planned comparison) but without correction for multiple comparisons. The data generated was analysed with GraphPad Prism v9.0.0.

### Quantification of EPO levels by ELISA

Human EPO levels in cell culture medium and from mouse serum samples was analysed using a human EPO Quantikine IVD ELISA kit (DEP00, R&D Systems, Abingdon, UK) following the manufacturer's instructions. Samples were diluted at different ratios (1 in 50 to 1 in 4000) using the sample diluent provided in the kit before analysis. The data generated was analysed using GraphPad Prism v9.0.0.

### Western blot

Western blot analysis was carried out using a rabbit anti-FLAG antibody (F7425, Merck, UK) and an IRDye® 800CW goat anti-rabbit secondary antibody (926-32211, LI-COR, UK). Plates containing HepG2 cells treated with LNPs were centrifuged at 500 rcf for 10 min at 4°C after which the supernatant was collected into a new plate. The protein content from mouse serum samples and *in vitro* cell supernatant was quantified using a BCA protein assay kit (23225, Thermo Scientific, UK) following the manufacturer's guidance. 50 μg of protein was loaded into each well of a 12-well NuPAGE^TM^ Bis-Tris mini gel under reducing conditions and run in MES SDS running buffer (NP0002, ThermoFisher Scientific, UK) for 35 min at 200 V. the gels were transferred unto a PVDF membrane (IB24002, ThermoFisher Scientific, UK) using an iBlot 2 dry blotting system. The membranes were left to dry in air and blocked using an Intercept® Blocking buffer (927-70001, LI-COR Biosciences, UK) for 1 h with shaking at 350 rpm at room temperature. The membranes were then incubated with the relevant primary antibodies at the recommended dilutions for 1 h at 350 rpm and room temperature after which they were washed thrice for 5 min with shaking. The membranes were subsequently incubated with the secondary antibody at 1:10 000 dilution for 1 h with shaking at 350 rpm and at room temperature after which they were washed four times with 1× TBS–Tween for 5 min with gentle shaking. The membranes were then imaged using an Odyssey® imager.

### Multiplex cytokine assay

Mouse serum cytokine levels were measured using an MSD (Meso Scale Diagnostics, Maryland, USA) multiplex assay kit which measures MIP-α, IP-10, IL-1β, IL-6, KC, IFN-γ and IL-5 following the manufacture's protocol. 25 μl of each serum sample was measured in duplicates using the Bio-Plex 200 system (Bio-Rad, Hertfordshire, UK) and the data plotted using Graph Pad Prism v9.0.0.

### Sample processing for mass spec analysis

200μl of serum per mouse was processed using an EPO enrichment column (Art. 1430, MAIIA Diagnostics, Uppsala, Sweden) following the manufacturer's instructions. Purified EPO protein was quantified by ELISA (DEP00, R&D Systems, UK) and trypsin digested using an in-solution tryptic digestion kit (89895, ThermoFisher Scientific, UK) following the manufacturer's instructions. Peptide digests were passed through a sample clean up step using a Sep-Pak C18 classic cartridge (WAT051910, Waters, UK) following the manufacturer's instructions. Peptides were eluted from the column with a buffer containing 50% ACN and 0.1% TFA in ultrapure water. Eluted peptides were dried under vacuum and reconstituted in 40 μl of 0.1% FA in ultrapure water for subsequent mass spec analysis.

### LC–MS method and pre-processing of data

A Waters nano-Acquity UPLC (M Class) with nano-ESI source and Thermo Scientific Q-Exactive HF-X LC/MS system were used for the separation and detection of peptides. 4ul sample was injected and trapped on a Waters C18 Trap column (5 μm, 2G 18 μm × 20 mm) for 3 min at 5 μl/min of 99% A (solvent A—0.1% formic acid in water). Samples were then separated across a Waters peptide BEH C18 analytical column (1.7 μm, 75 μm × 250 mm), with a flow rate of 0.3 μl/min over a 120 min gradient from 3–35% B (solvent B—0.1% formic acid in acetonitrile). Data was acquired on a Thermo Q-Exactive HF-X, operating in positive ion mode. Full MS was acquired over 350–2000 *m*/*z* scan range with resolution set to 120 000 and AGC target at 3 000 000 with maximum accumulation time of 100 ms. For ddMS2, system was set to select the Top 10 (most abundant) precursors in 1.2 *m*/*z* wide isolation window at normalized collision energy of 30. AGC was set at 100 000 with maximum accumulation time of 50ms. Data generated was analysed using Qual Browser (Thermo XCalibur 3.0). Peptides of interest were manually searched in total ion chromatogram (TIC) and precursor ions was extracted for each peptide of interest. Peaks were manually integrated to obtain the peak area and intensity.

## Results

### Design and development of PALS

PALS was designed to assess the hypothesis that a pool of LNPs administered to test animals can reliably determine the functionality of individual LNPs with a similar outcome as when the LNPs are administered individually to groups of animals. To test this hypothesis, we answer three key questions: (i) will barcoded mRNA be efficiently translated to functional barcoded proteins? (ii) Can the barcode measurement by mass spectrometry be used as a surrogate for protein quantification? (iii) Is PALS sensitive enough to quantify and differentiate LNPs in a pool?

**Figure 2.**
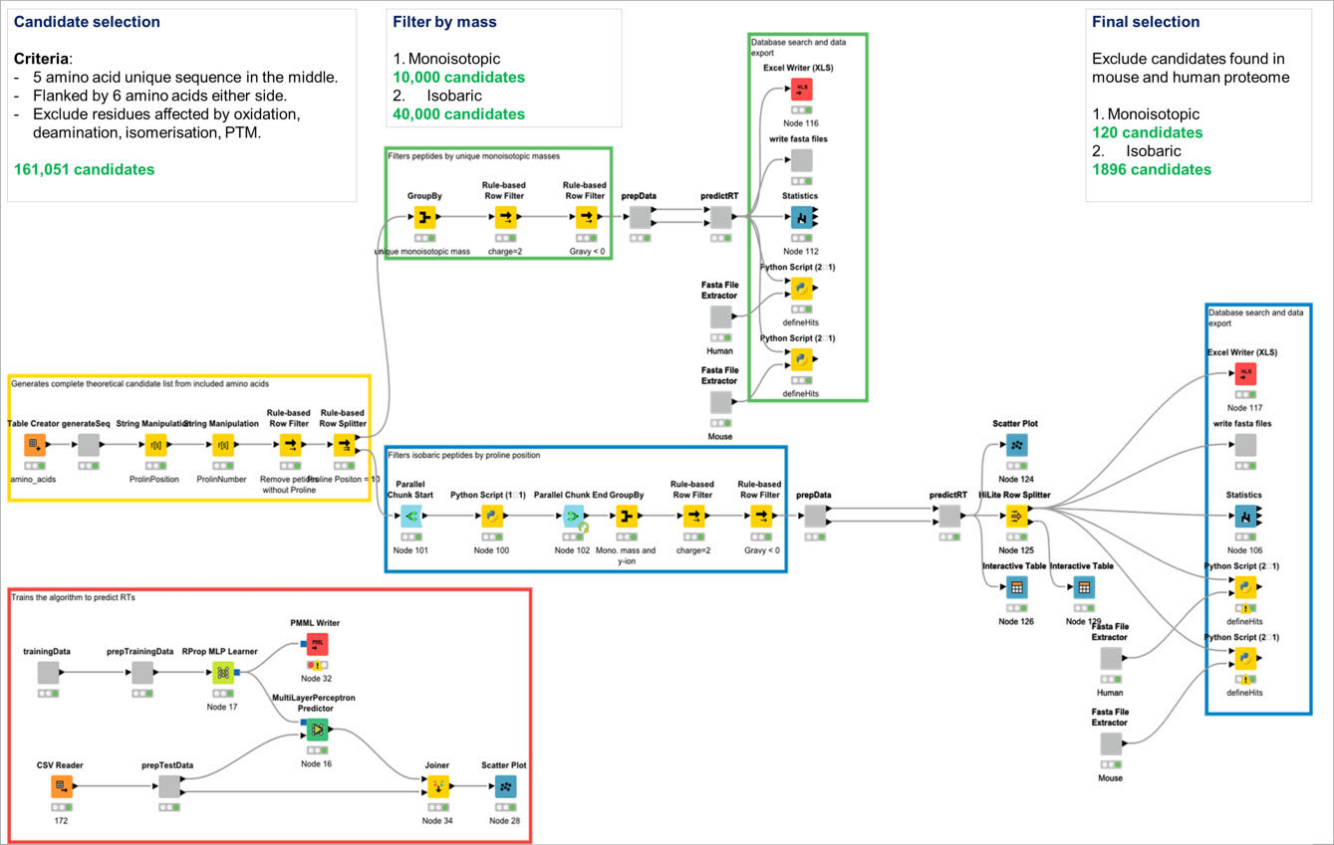
Data driven design of peptide barcode sequences. Peptide barcode candidates were designed and selected using KNIME following stringent design criteria

To begin with, the barcodes were designed so that only the five amino acids in the middle of the sequence were randomized. Flanking regions containing six amino acids were included on each side of the trypsin cleavage sites to facilitate consistent trypsin digestion efficiency (18) (Figure 1B). The design was such that on ionization, precursor ions of specific mass to charge (*m*/*z*) can be selected (MS1) from a mixture of ions and later fragmented in a collision cell to produce the product ions (MS2) for each peptide. Specific residues were excluded (M,W,N,Q,C,R,K,D,E) from the barcode sequence to minimize the impact of any post-translational modifications and therefore loss of signal upon analysis by mass spectrometry. A proline residue was included in all barcodes to aid identification by MS2 and act as a diagnostic fragment ion. Once the list of residues was confirmed, a script was written using the KNIME analytics platform (19) (KNIME, Germany) to generate a database of all possible barcodes. This initial database (>161 000 candidates) was then filtered using rule-based filters to first include only those which had prolines in the variable region of the barcode (50 000 candidates). Once narrowed down, a final filter was used to identify barcodes which had unique monoisotopic masses, so that they could be resolved at both the MS1 and MS2 levels (120 final candidates). The final filter also provides a library of 1896 isobaric peptide candidates which could be applied in a larger screen of >120 LNPs (Figure 2). The barcodes were subsequently built into a library of DNA vectors containing these barcodes encoded for at the 3′ end of the open reading frame (ORF) of human erythropoietin and Cre recombinase. Each barcoded mRNA was formulated into a unique test LNP and up to 10 (9) barcoded LNPs (bLNPs) were pooled together and administered to a test group of mice. The barcoded protein was extracted from the serum of treated mice using an EPO purification kit (Art.1390, MAiiA Diagnostics, Uppsala, Sweden) and enzyme digested to release the barcode peptides which were then quantified by mass spectrometry (Figure 1A). The relative quantitation of the barcode peptides was representative of the functionality of the bLNPs.

### Barcoded mRNA is efficiently translated into functional barcoded proteins

**Figure 3.**
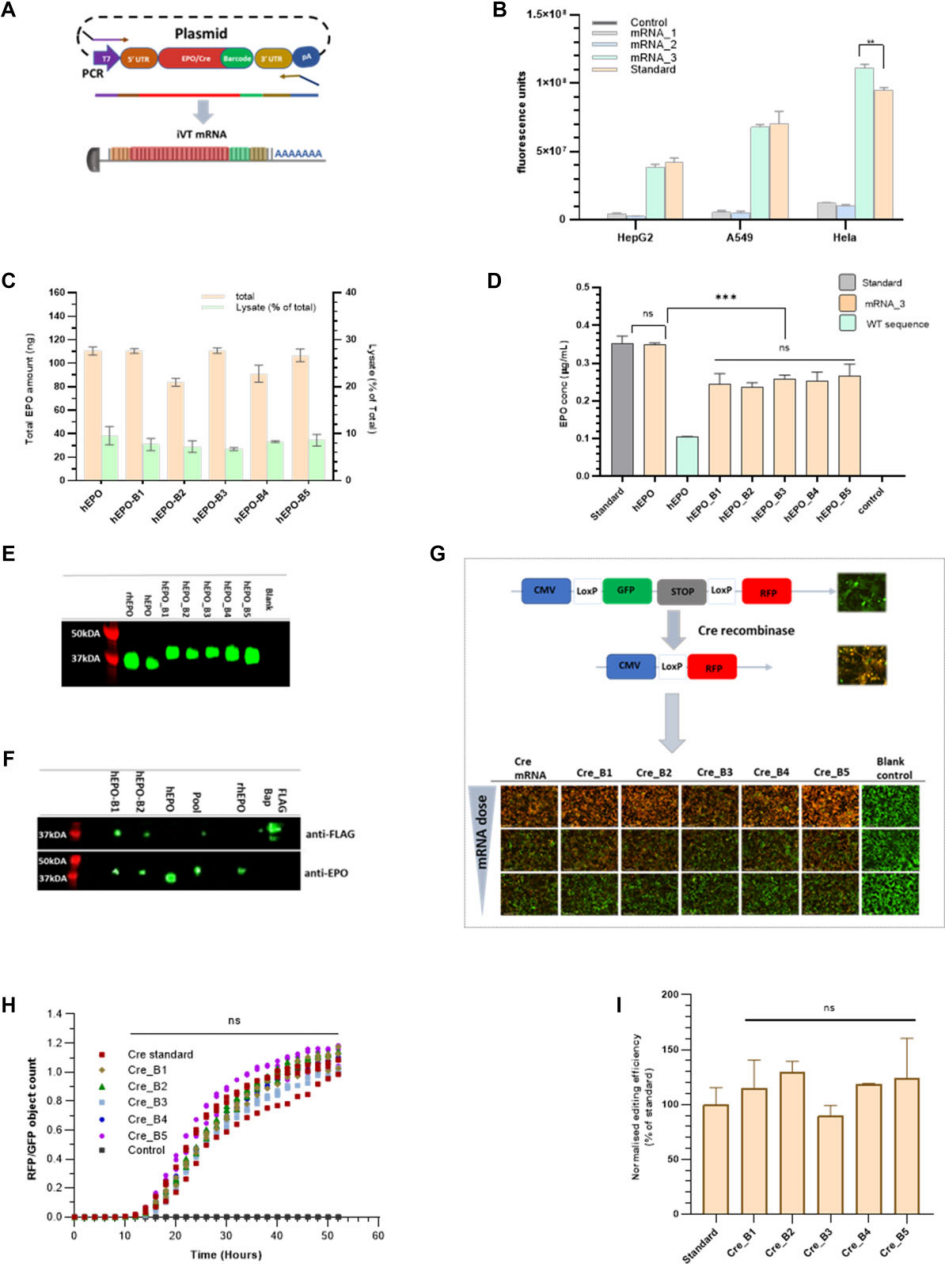
Barcoded mRNA is functional and efficiently translated to functional barcoded proteins. (**A**) Representation of the process for *in vitro* transcription of bmRNA. (**B**) Effect of optimization of UTRs and Poly A tail on the translation efficiency of eGFP mRNA in different cell lines. Values are mean ± SD, *n* = 3. (**C**) Effect of optimization of hEPO ORF on the translation efficiency of hEPO and bhEPO mRNA in Hep G2 cells. Values are mean ± SD, *n* = 3. (**D**) Hep G2 cells show comparable translation and secretion of EPO proteins following treatment with the same molar unit of mRNA. Values are mean ± SD, *n* = 2. (**E**) Western blot showing bmRNA is fully translated into barcoded protein with a slightly higher molecular weight than the non-barcoded protein. (**F**) Western blot showing pooled bmRNA is fully translated into barcoded proteins *in vivo* and can be detected by both anti-FLAG and anti-EPO antibodies. (**G**) Representation of the colour-switch HEK 293 Cre reporter cell line cassette. Cells express green fluorescent protein (GFP), giving off a green signal but express red fluorescent protein (RFP) when successfully edited by Cre recombinase. (**H**, **I**) Quantitation of ratio of RFP count to GFP count in HEK 293 LoxP- GFP-RFP cells dosed with 400 ng mRNA and expressed as normalised means ± SD, with *n* = 3. Data presented as a time course (H) and normalized editing efficiency of bmRNA relative to the standard 48 h after transfection (I). Statistical significance determined by one-way ANOVA

Because the barcodes are encoded for at the C-terminal the ORF or the reporter protein, we anticipated that the barcodes could affect the translation efficiency and functionality of the barcoded mRNA, potentially limiting the size of each LNP pool. To mitigate this, we optimized the parent DNA vectors for efficient mRNA translation (Figure 3A) compared with a standard commercial non barcoded mRNA from TriLink®. Specifically, we designed the ORF codon of the mRNA sequence to be approximately 62% rich in GC bases and sourced this through GeneArt®, optimised the UTRs sequences as well as the length of the PolyA tail and tested these iterations *in vitro* using different cell lines. First, we optimized the UTRs and poly A tail length using a non-barcoded eGFP mRNA ORF and observed the impact on translation in three different cell lines: A549, HeLa and HepG2. We tested three combinations of UTRs and Poly A tail: mRNA_1 consisting of an albumin 3′UTR, a HSD 5′ UTR and 75 Adenosine base tail; mRNA_2 consisting of an albumin 3′UTR, a HSD 5′ UTR and a poly A tail stem loop containing 40 Adenosines and 30 Cytosine bases and mRNA_3 consisting of a synthetic 5′UTR, a codon optimised β-haemoglobin 3′ UTRs and a Poly A tail consisting of 120 Adenosine bases. We found mRNA_3 to be comparable to the reference standard in the three cell lines tested (Figure 3B). We proceeded to optimize the ORF of human erythropoietin using the same vector backbone as mRNA_3 and synthesized a library of vectors containing barcoded hEPO using the GeneArt® service. Figure 3C shows that translation from bmRNA was comparable between different barcodes, producing about 250% more protein than the wild type non-barcoded mRNA sequence.

Next, we questioned whether the barcodes might affect the secretion of hEPO, resulting in the relatively lower amount of quantified hEPO in the cell supernatant. We evaluated this by normalizing the amount of protein produced to the molar amount of mRNA dosed and quantifying the total amount of hEPO in each well, that is, both intracellular and secreted. We observed that a comparable amount of protein was produced per molecule of mRNA, approximately 10% of which was intracellular for both barcoded and non-barcoded mRNA (Figure 3d). This shows that the inclusion of the barcodes c-terminally does not significantly reduce the translation efficiency of the mRNA, neither does it result in hEPO splicing or an alteration of the hEPO secretory signal. Since the secretion of EPO is usually preceded by the cleavage of a 27 amino acid N-terminal leader sequence and the C-terminal arginine (20), we sought to determine if the bhEPO mRNA was fully translated and contained the barcode peptides. Western blot analysis shows that bhEPO mRNAs were translated into barcoded hEPO proteins *in vitro*, migrating at about 40 kDa compared with the recombinant hEPO standard and the non-barcoded hEPO protein translated from a non-barcoded mRNA which both migrated at about 35kDa (Figure 3E). Furthermore, western blot analysis of *in vivo* expressed bmRNA individually and in a small pool consisting of two bmRNAs also confirmed full translation of the protein with both anti-EPO and anti-FLAG antibodies (Figure 3F). Next, we analysed serum samples obtained from mice which had received a pool of bhEPO mRNA. We identified all five barcodes as well as representative hEPO peptides: LYTGEACR, VYSNFLR, YLLEAK, TITADTFR, EAISPPDAASAAPLR (Supplementary figure S1), confirming that bhEPO mRNA is fully translated into bhEPO proteins and these proteins were efficiently secreted both *in vivo* and *in vitro*.

Next, we sought to evaluate this approach on a functional protein. We chose to use a HEK 293 colour-switching CRE reporter cell line which expresses a LoxP-GFP-stop-LoxP-RFP cassette under a CMV promoter to test the robustness of this approach. The cells express the GFP signal when CRE protein is not present and the RFP signal once the DNA fragment between the two LoxP sites has been deleted by CRE (Figure 3G) We synthesized barcoded Cre mRNA (bCre) using the same previously optimised DNA vector backbone and transfected the HEK 293 dual reporter cells with these bCre mRNAs. Once translated, the bCre proteins migrate into the nucleus to delete the DNA fragments between the two LoxP sites. The bCre mRNA transfected cells showed comparable gene editing efficiency to the non-barcoded Cre mRNA standard with (Figure 3H, I). Collectively, the data shows that the barcodes do not alter the translation and functionality of the mRNA.

### PALS is a good surrogate for ELISA-based protein quantitation

**Figure 4.**
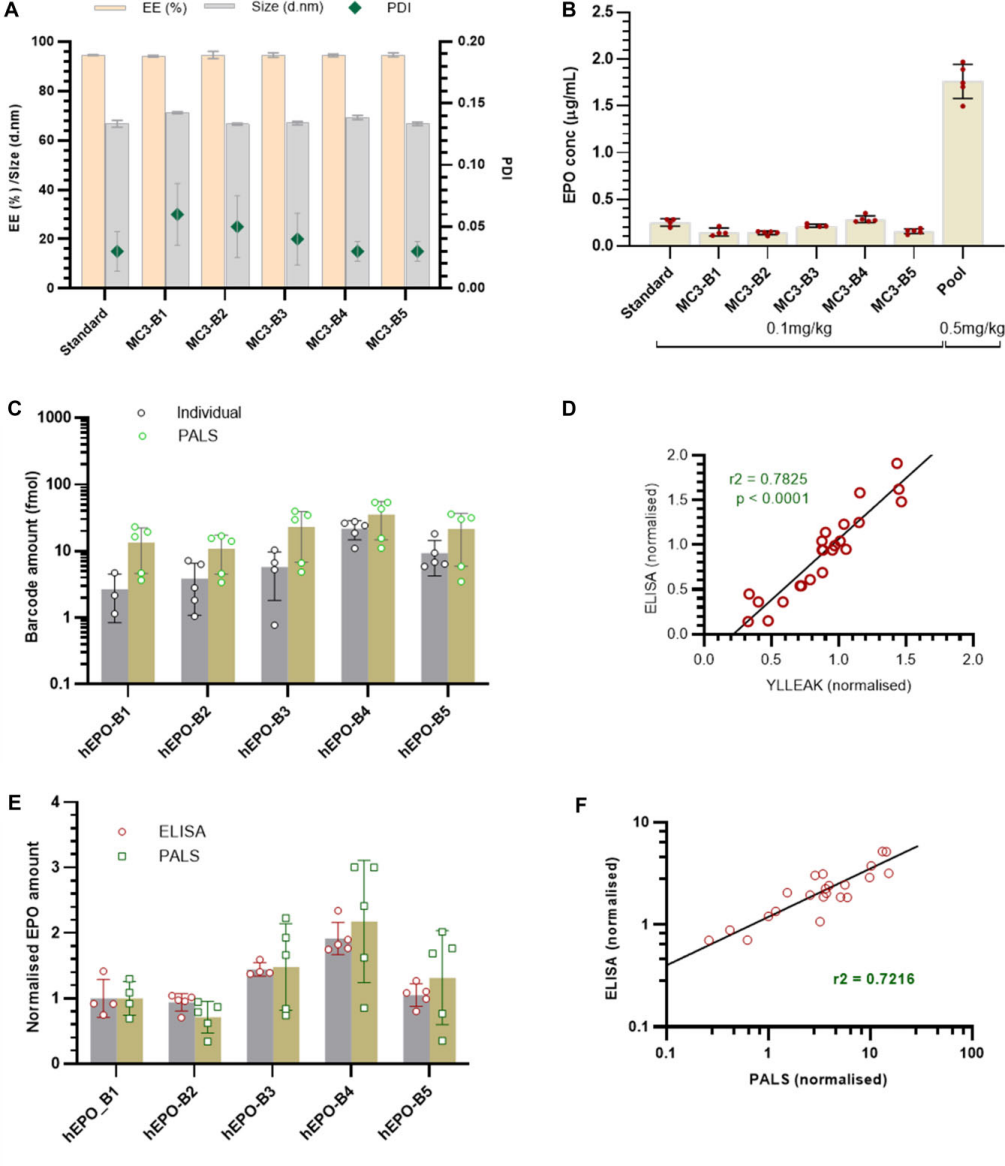
PALS is a good surrogate for ELISA-based protein quantitation. (**A**) LNP particle characteristics including size, polydispersity index (PDI) and encapsulating efficiency. Values are mean ± SD, *n* = 5. (**B**) Quantitation of bhEPO amounts in mouse serum 6 h after LNP administration *in vivo*. Values are mean ± SD, *n* = 5. (**C**) quantitation of bhEPO using barcodes after single LNP administration and using PALS. No difference in ranking of LNPS based on barcode amounts between both methods (Wilcoxon matched pairs signed rank test, *P*= 0.0625). (**D**) Correlation between EPO protein quantification by ELISA and by mass spectrometry using one representative EPO peptide. (**E**) comparison of protein quantitation using ELISA and PALS. No difference in normalized mean between both methods (paired *t*-test) and no difference in ranking of LNPs using both methods (Wilcoxon matched-pairs signed rank test, *P*= 0.3750). (**F**) correlation of protein quantitation by ELISA and PALS using the peptide barcodes.

Critical to the application of PALS is the ability to screen for functional delivery of LNPs. For individual treatment of LNPs, this is routinely done by examining functional read-outs such as protein expression, fluorescence, luminescence, or INDELS. None of these methods however permit direct correlation of the phonotypic signals to individual treatments in a large pool. To assess the accuracy of PALS in directly linking a phenotypic signal to a particular type of LNP in a pool, we sought to compare the functional read-out from PALS to a gold standard for protein quantitation: ELISAs. We formulated five bhEPO mRNA into very similar LNPs. LNP characteristics were not statistically different for the five bLNPs with sizes ranging from 66nm to 70nm and mRNA encapsulation efficiency of 94–95% (Figure 4A). We administered each bLNP to a group of female Balb/C mice at a bmRNA dose of 0.1 mg/kg and another group of mice was administered a pool of all five bLNPs at a dose of 0.1 mg/kg/bLNP (total bmRNA dose of 0.5 mg/kg). We observed the mean bhEPO amount in individual groups measured by ELISA was about 10% of the bhEPO amount in the pool, which is an indication that pooling the LNPs did not result in a decrease in total bhEPO production (Figure 4B). We further processed the serum samples and quantified each barcode as well as an EPO peptide YLLEAK. Each barcode was quantified from the mice dosed with the pool of bLNPs. A higher amount of each barcode was quantified from the pool than from the individual groups, corresponding to the higher total protein amount from the pool observed with the ELISA measurement (Figure 4C). The quantification of each bLNP, normalised to the hEPO amount from the standard LNP treated group, was well correlated between both methods of quantitation (*r*^2^ = 0.7825) (Figure 4D). We also observed that when normalized against protein amounts from hEPO_B1, there was no difference in the normalized means of each bLNP, the same fold change relative to hEPO_B1 was obtained and the ranking of each bLNP was consistent for PALS as for ELISA measurement (Figure 4E) with a correspondingly good correlation in normalized protein quantitation between both methods (Figure 4F).

### PALS is sensitive to differentiate LNPs in a pool

**Figure 5.**
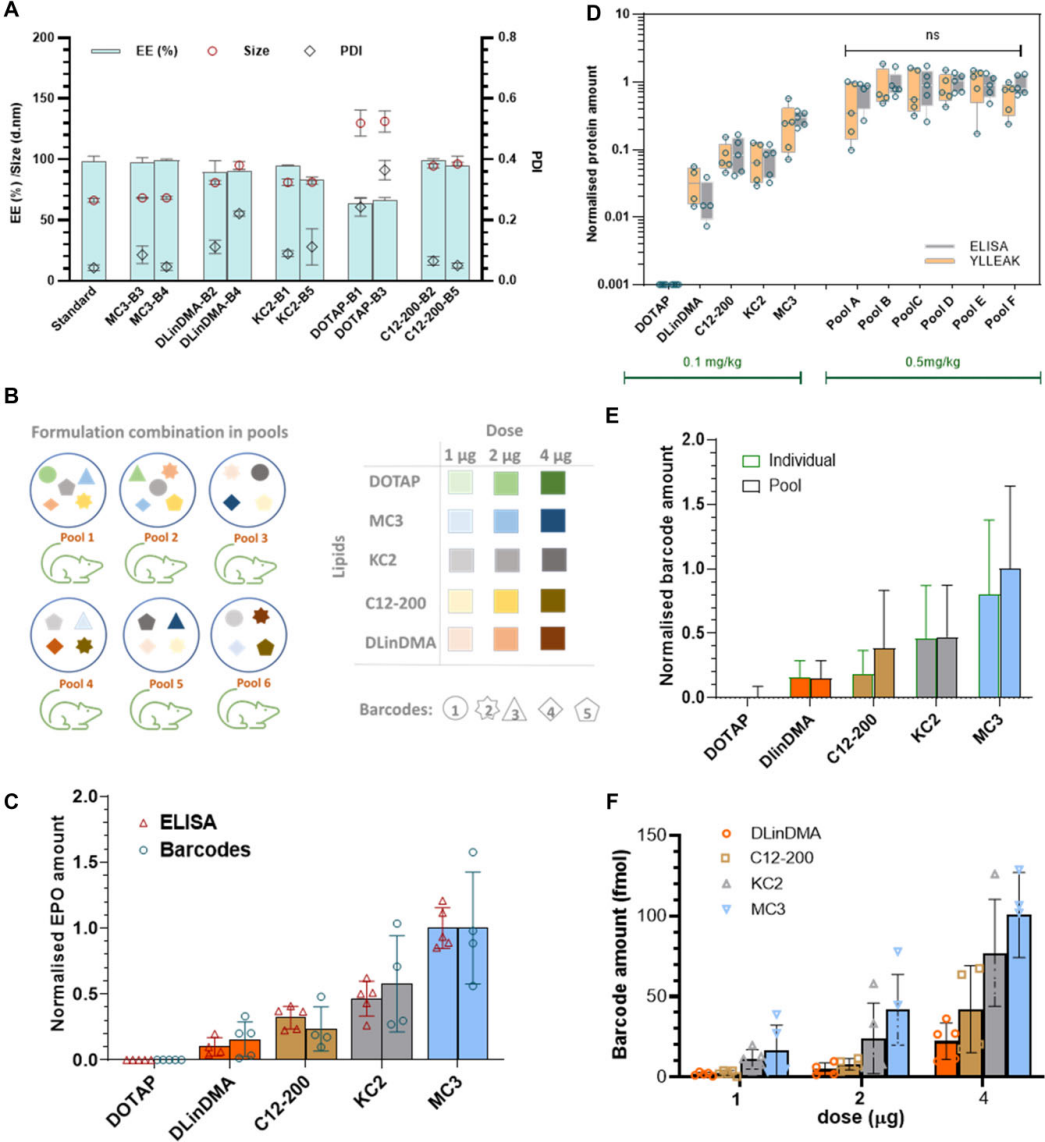
PALS is sensitive to differentiate LNPs and rank them based on functionality. (**A**) LNP particle characteristics including size, polydispersity index (PDI) and encapsulating efficiency. Values are mean ± SD. (**B**) representation of bLNP combinations in each pool. Total mRNA dose per pool was 10 μg. (**C**) Quantification of EPO protein from mouse serum 6 h after i.v. administration measured by ELISA and mass spectrometry normalized to the standard LNP control. Values are mean ± SD, *n* = 5. (**D**) Quantification of hEPO in the individual LNP treated groups is consistent with quantification using the barcodes with a good correlation between both methods. Values are mean ± SD, *n* = 5. (**E**) Quantification of barcodes in the animals which received a single LNP is consistent with quantification of barcodes in animals which received the pool. Values are mean ± SD, *n* = 5. (**F**) Dose-response for each bLNP showing PALS is sensitive to differentiate and rank LNPs based on functionality at different dose levels. Values are mean ± SD, *n* = 5.

The relationship between the dose of hEPO mRNA and the amount of protein expressed *in vivo* has been shown to not be linear (21) and the relative hEPO production from different LNPs could be different at different doses (5). To this end, we sought to determine how sensitive PALS is in differentiating and ranking different LNPs in a pool containing different LNPs with varied efficacy. We formulated five bhEPO mRNAs into LNPs using different well-studied ionisable lipids (Supplementary figure S2a): DLIn-MC3-DMA, DLin-KC2-DMA, DOTAP, DLin-DMA and C12-200 (22–24). All LNPs were formulated using standard components as described in Supplementary figure S2b, but with different ionizable lipids bmRNAs. Figure 5A shows the characteristics of the formulated bLNPs and the differences observed were within the ranges expected for particles made with these cationic lipid components. Each ionisable lipid was used to formulate two different bmRNAs with similar size and encapsulation efficiency. To explore the relationship between dose and protein expression, we administered 0.1 mg/kg bLNPS to individual groups of mice and prepared different pools of bLNPS containing a mixture of bLNPS at different dose levels: 0.05 mg/kg/bLNP, 0.1 mg/kg/bLNP and 0.2 mg/kg/bLNP. The total mRNA dose per pool was maintained at 0.5 mg/kg (Figure 5B). We selected the lowest dose level of 0.05 mg/kg/bLN, 1/10th of a 0.5 mg/kg total mRNA dose based on the total protein expression levels expected at 0.5 mg/kg and to be within the limits of protein quantitation (Supplementary Table S1). We kept the maximum total mRNA dose level at 0.5 mg/kg to minimize potential safety risks.

As was observed previously with pooled MC3 bLNPs, the quantitation of the total bhEPO was consistent between PALS and ELISA with no significant difference in the normalized mean amounts measured using both methods (Figure 5c). PALS also provided the same ranking of each bLNP based on the efficacy of the ionizable lipids as observed using ELISA as thus: MC3 > KC2 > C12-200 > DLin-DMA > DOTAP (Figure 5D) and with an excellent correlation between both methods (*r*^2^ = 0.9699) (Supplementary figure S2c), which shows that PALS is predictive of protein measurement by ELISA. Furthermore, at the same dose of bmRNA, there was no difference in the barcode amount quantified by PALS between the individual groups and the pools for each ionizable lipid (Figure 5E). We subsequently quantified the barcodes from the pooled groups which received different doses of bLNPs and observed a corresponding dose response for each bNLP which preserves the ranking of each bLNP at the different dose levels (Figure 5F). We also note that the dose response for each bLNP formulation made with a particular cationic lipid and the corresponding bmRNA cargo is characteristically different as expected (5), showing that the functional peculiarities of each bLNP is preserved in a pool and measurable by PALS. Remarkably, we note that treating mice with a pool of different bLNPs does not result in elevated cytokine levels at a standard total LNP dose (Supplementary figure S3), showing that PALS is capable of safely differentiating bLNPs at 1/10th the standard LNP dose in a pool.

**Figure 6.**
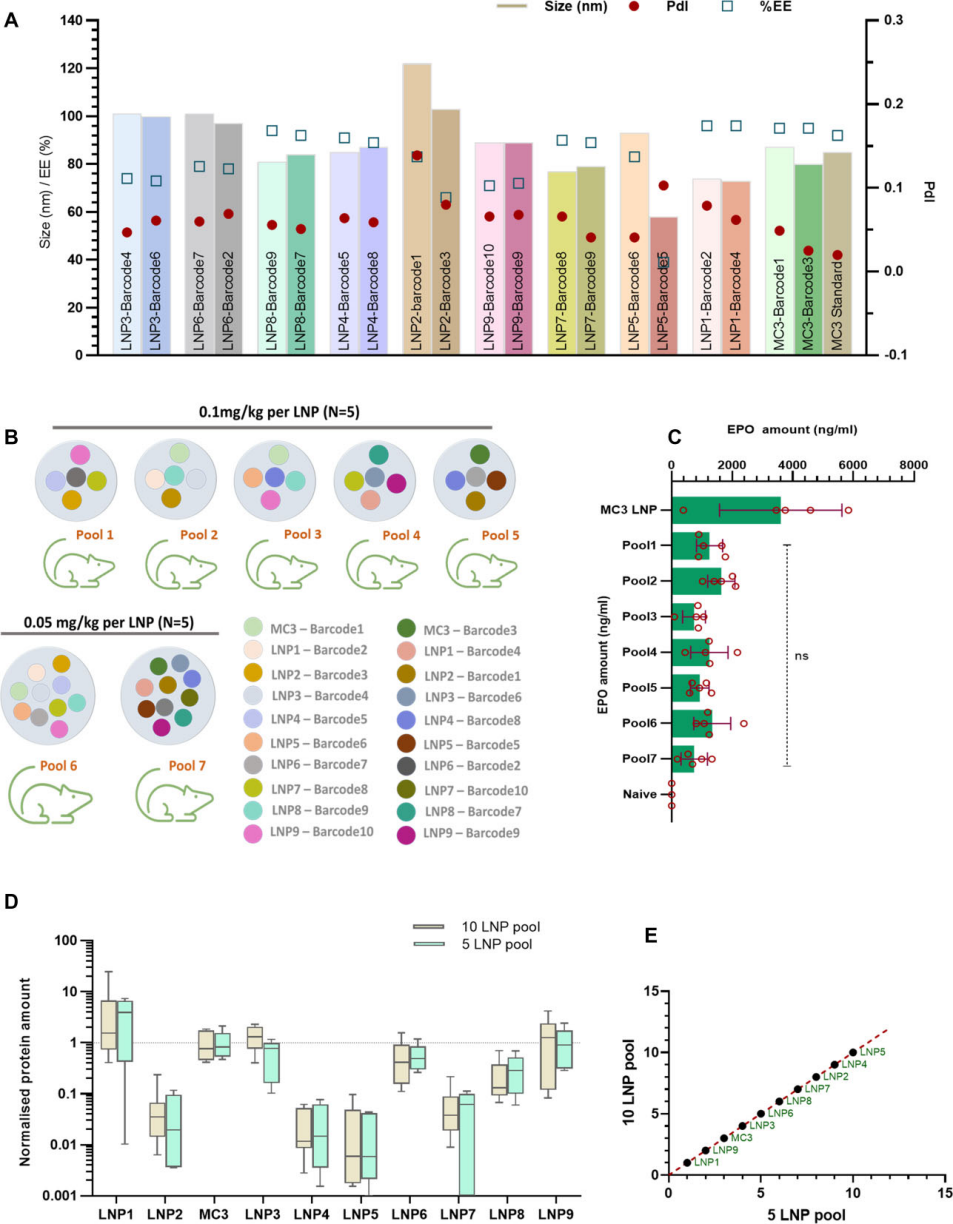
PALS enables lead LNP identification from a pool of 10 LNPs. (**A**) Formulation and characterisation of 10 novel LNPs using 10 unique bmRNAs. Each novel LNP was formulated with two different bmRNAs for comparison. (**B**) bLNP combination in different pools. Female Balb/C mice were dosed with five (5) or ten (9) LNP pools at a total mRNA dose of 0.5 mg/kg. (**C**) Quantification of hEPO protein from the treated mice by ELISA. No difference in mean protein expression between the different pools. Values are mean ± SD, *n* = 5. (**D**) Normalized expression of protein from each bLNP was similar between a 5LNP pool and a 10LNP pool. Values represent mean ± SD of a protein expression relative to the MC3 standard LNP. (**E**) Ranking of bLNPs from a pool of 10 LNPs is consistent with ranking from a pool of five LNPs

We subsequently examined the accuracy of PALS in differentiating and ranking bLNPs in a pool of 10 LNPs compared to a pool of five LNPs. To do this, we carried out a screen of nine novel LNPs, including a standard MC3 LNP as a control. Each lipid was used to formulate two different bmRNAs for comparison and Figure 6A shows that bLNPs formulated with the same cationic lipids have similar physical characteristics. The bLNPs were then pooled in different combinations as illustrated in Figure 6B. Groups of female Balb/C mice were administered a pool of five (5) unique bLNPS at an mRNA dose of 0.1mg/kg/bLNP or ten (9) unique bLNPs at a dose of 0.05 mg/kg/bLNP. Both groups of mice received a total mRNA dose of 0.5 mg/kg. There was no difference in the total protein levels measured by ELISA between the pooled groups (Figure 6C). As was expected, a lower barcode amount was measured from the pool of 10 LNPs compared with the pool of five LNPs, reflecting a dose response (Supplementary figure S4). When normalized against an MC3 bLNP, there was no difference in the normalised protein amount measured by PALS between the five LNP pool and the 10 LNP pools (Figure 6D). In addition, the ranking of each bLNP in a pool of five LNPs was well correlated with the ranking in a pool of 10 LNPs (Figure 6E). Collectively, this shows that PALS is sensitive to safely differentiate and accurately rank up to 10 LNPs in a pool based on the functionality of the LNP and the mRNA cargo.

## Discussion

*In vivo* screening of LNPs is typically cost and time intensive, requiring the use of a large number of experimental animals. It is however an essential part of the drug development process which is required because of the inadequacy of *in vitro* screening methods for accurately predicting efficacy of LNP formulations *in vivo* (4,25). Approaches to enable the high throughput screening of LNPs *in vivo*, including the use of DNA barcodes are limited in their ability to accurately assess functional delivery of LNPs since they rely on the quantification of a separate DNA molecule which is not translated (6,7). DNA barcodes are valuable for screening a very large number of nanoparticle combinations due to the advantage of signal amplification inherent with deep sequencing. However, this advantage is less relevant for a screen of clinically relevant cargos. This is because the DNA barcodes are not therapeutically relevant and the dose of each individual LNP in such a large pool might be too low to assess therapeutic efficacy in the same screen. Thus, an *in vivo* screening platform which enables the direct assessment of the functional delivery of functional RNA cargo in a clinically relevant context offers an advantage over the use of DNA barcodes. We consider that an ideal system will be model, cargo and delivery system agnostic, allowing for the integration of as many parameters as possible to reduce the size and frequency of *in vivo* studies. We have described the development of a proteomics-based system where in barcode sequences are incorporated in the 3′ end of the ORF of a relevant mRNA which is translated into a unique barcoded protein. The barcoded proteins are extracted from tissues and analysed using LC-MS/MS and the relative quantitation of each peptide barcode used to assess the functionality of the LNPs.

Two critical considerations in designing the barcoded mRNA are the effect of the barcode sequence on the translation of the mRNA and the effect of the barcode peptide extension on the functionality of the translated protein. Because each barcoded protein is a unique protein variant, we designed the barcode extensions to be as similar as possible to minimize translational and functional differences and thus preserve differences associated with the delivery system. Consequently, we designed the barcodes to have consistent flanking regions on each side of a variable core. This ensured that the mRNA sequences were largely similar and further served to ensure homogenous cleavage of the barcoded proteins during processing for MS analysis (18). Furthermore, given that large fusion proteins have been shown to be translated efficiently from nucleic acids and remain functional (26,27), we considered that a C-terminal extension <10% the molecular mass of the protein will have little effect on the structure and function of the translated protein.

Some limitations to the use of PALS include the challenge of accurately assessing safety events attributable to specific LNPs in a pool. While the dose of each individual LNP in a pool is very low and perhaps below levels which might trigger an inflammatory response, it is important to be certain that such events do not occur or influence the outcome of the screen. An initial *in vitro* screen might help with identifying inflammatory LNPs which could be excluded from the *in vivo* study. PALS is also limited by the amount of protein that can be produced *in vivo*. The lower limit of Quantitation (LOQ) for PALS about 2 fmol/μl for each peptide barcode (Supplementary Table S1) which translates to approximately 0.6 ng/mL of a 30kDa protein. Thus, a pool of 10 LNPs should yield a total protein amount of approximately 6 ng/ml assuming 100% recovery after sample processing. The pool size can thus be increased or decreased depending on the expected total protein yield and the difference in expected relative efficacy of each LNP in the pool. These constraints imply that PALS not applicable where protein levels achieved from exogenous mRNA is expected to be very low or undetectable as in the case of vaccines.

We assessed the application of this system using two models, representative of different therapeutic applications. First, we used a secreted protein hEPO as a proof of concept to evaluate the sensitivity and specificity of PALS in screening multiple LNPs for application in antibody and protein replacement therapies (28). We observed that the barcode extensions did not significantly alter the translation or secretion of hEPO, neither did it alter the binding of hEPO to antibodies in both ELISA and western blot assays. We did not assess the effect of the barcodes on reticulocyte count or % haematocrit which are measures of the functionality of the hEPO protein (29) since the purpose of our screen was assessment of the functionality of the LNP and the cargo. Based on our data, we propose that PALS can be applied in screening lipids (and other delivery systems) as well as the RNA cargo for secreted protein applications where the purpose of the screen is assessing the functionality of the delivery system or the effect of RNA structural modifications on protein levels. For other applications including intracellular proteins and gene editing, we used a Cre reporter model to assess the effect of the barcodes on the functionality of the barcoded protein *in vitro*. We observed that the barcodes do not alter the genome editing efficacy of the translated Cre protein. We propose thus that functional assays can be integrated into PALS to enable a multi-parameter assessment of the delivery system, cargo iterations and therapeutic effect of either.

Another potential application for PALS is to screen for cell and tissue specific distribution of potential therapeutics. We propose that using deep sequencing, the barcode sequences in the mRNA can be quantified in specific cell populations and directly correlated with the peptide barcode signals measured from those. Critical to this application is the ability to recover sufficient individual barcoded protein amounts from each tissue/cell type. A recent publication demonstrated this potential using peptide barcode tagged monomeric streptavidin (mSA) to screen a library of 384 ionizable lipids in pool sizes of 48 LNPs by quantifying the barcode peptides in mouse liver tissues (15). The authors selected mSA as the carrier protein and used an myc tag to achieve optimal peptide enrichment from the liver tissues. Distribution to specific cell types within the liver and tissues in other organs is an opportunity which remains to be explored.

To conclude, we have presented a design for peptide barcoding which enables a more efficient screen of LNPs and potentially other nucleic acid delivery systems. We show that the barcoded mRNA is efficiently translated into barcoded proteins which remain functional, and the relative amount of each peptide barcode accurately represents the relative efficacy of the associated LNP. Indeed, the apparent differences in rate of translation did not affect the ranking of LNPs in a pool. We demonstrated that we could administer up to 10 LNPs in a pooled dose to mice and obtain the same outcome as if we administered the same 10 LNPs individually, despite the variations due to mRNA translation and the relative dose-dependency of LNP efficacy. Furthermore, we show that PALS does not induce toxicity and in fact, is a safe way to screen for the efficacy of multiple LNPs at a fraction of the dose, cost, time and lab animals used.

## Supplementary Material

gkae648_Supplemental_File

## Data Availability

The data underlying this article are available in the article and in its online supplementary material. Mass spectrometry data have been deposited to the ProteomeXchange Consortium via the PRIDE partner repository with the dataset identifier PXD053797.

## Notes

Present address: Surender Vashist, NeoVac Ltd, Unit 1&2 West, 127 Olympic Avenue, OX14 4SA, Milton Park, UK.

Present address: Arpan Desai, CamGene Therapeutics, The Mill SciTech Park, Mill Lane, Hauxton, Cambridge CB22 5HX, UK.

## References

[1] Gillmore J.D. , Gane E., Taubel J., Kao J., Fontana M., Maitland M.L., Seitzer J., O’Connell D., Walsh K.R., Wood K. et al. CRISPR-Cas9 in vivo gene editing for transthyretin amyloidosis. N. Engl. J. Med. 2021; 385:493–502. 34215024 10.1056/NEJMoa2107454

[2] Adashi E.Y. , Gruppuso P.A., Cohen I.G. CRISPR therapy of sickle cell disease: the dawning of the gene editing era. Am. J. Med. 2024; 135:390–392. 10.1016/j.amjmed.2023.12.018 38184185

[3] Lokugamage M.P. , Vanover D., Beyersdorf J., Hatit M.Z.C., Rotolo L., Echeverri E.S., Peck H.E., Ni H., Yoon J.-K., Kim Y. et al. Optimization of lipid nanoparticles for the delivery of nebulized therapeutic mRNA to the lungs. Nat. Biomed. Eng. 2021; 5:1059–1068. 34616046 10.1038/s41551-021-00786-x PMC10197923

[4] Paunovska K. , Sago C.D., Monaco C.M., Hudson W.H., Castro M.G., Rudoltz T.G., Kalathoor S., Vanover D.A., Santangelo P.J., Ahmed R. et al. A direct comparison of in vitro and in vivo nucleic acid delivery mediated by hundreds of nanoparticles reveals a weak correlation. Nano Lett. 2018; 18:2148–2157. 29489381 10.1021/acs.nanolett.8b00432 PMC6054134

[5] Cheng Q. , Wei T., Farbiak L., Johnson L.T., Dilliard S.A., Siegwart D.J. Selective organ targeting (SORT) nanoparticles for tissue-specific mRNA delivery and CRISPR–Cas gene editing. Nat. Nanotechnol. 2020; 15:313–320. 32251383 10.1038/s41565-020-0669-6 PMC7735425

[6] Dahlman J.E. , Kauffman K.J., Xing Y., Shaw T.E., Mir F.F., Dlott C.C., Langer R., Anderson D.G., Wang E.T. Barcoded nanoparticles for high throughput in vivo discovery of targeted therapeutics. Proc. Natl. Acad. Sci. U.S.A. 2017; 114:2060–2065. 28167778 10.1073/pnas.1620874114 PMC5338412

[7] Sago C.D. , Lokugamage M.P., Paunovska K., Vanover D.A., Monaco C.M., Shah N.N., Castro M.G., Anderson S.E., Rudoltz T.G., Lando G.N. et al. High-throughput in vivo screen of functional mRNA delivery identifies nanoparticles for endothelial cell gene editing. Proc. Natl. Acad. Sci. U.S.A. 2018; 115:E9944–E9952. 30275336 10.1073/pnas.1811276115 PMC6196543

[8] Viger-Gravel J. , Schantz A., Pinon A.C., Rossini A.J., Schantz S., Emsley L. Structure of Lipid Nanoparticles Containing siRNA or mRNA by Dynamic Nuclear Polarization-Enhanced NMR Spectroscopy. J. Phys. Chem. B. 2018; 122:2073–2081. 29332384 10.1021/acs.jpcb.7b10795

[9] Guimaraes P.P.G. , Zhang R., Spektor R., Tan M., Chung A., Billingsley M.M., El-Mayta R., Riley R.S., Wang L., Wilson J.M. et al. Ionizable lipid nanoparticles encapsulating barcoded mRNA for accelerated in vivo delivery screening. J. Controlled Release. 2019; 316:404–417. 10.1016/j.jconrel.2019.10.028 PMC7032071 31678653

[10] Boehnke N. , Straehla J.P., Safford H.C., Kocak M., Rees M.G., Ronan M., Rosenberg D., Adelmann C.H., Chivukula R.R., Nabar N. et al. Massively parallel pooled screening reveals genomic determinants of nanoparticle delivery. Science. 2022; 377:384–397. 10.1126/science.abm5551 PMC10249039 35862544

[11] Dobrowolski C. , Paunovska K., Schrader Echeverri E., Loughrey D., da Silva Sanchez A.J., Ni H., Hatit M.Z.C., Lokugamage M.P., Kuzminich Y., Peck H.E. et al. Nanoparticle single-cell multiomic readouts reveal that cell heterogeneity influences lipid nanoparticle-mediated messenger RNA delivery. Nat. Nanotechnol. 2022; 17:871–879. 35768613 10.1038/s41565-022-01146-9 PMC11784293

[12] Wittrup A. , Ai A., Liu X., Hamar P., Trifonova R., Charisse K., Manoharan M., Kirchhausen T., Lieberman J. Visualizing lipid-formulated siRNA release from endosomes and target gene knockdown. Nat. Biotechnol. 2015; 33:870–876. 26192320 10.1038/nbt.3298 PMC4663660

[13] Maugeri M. , Nawaz M., Papadimitriou A., Angerfors A., Camponeschi A., Na M., Hölttä M., Skantze P., Johansson S., Sundqvist M. et al. Linkage between endosomal escape of LNP-mRNA and loading into EVs for transport to other cells. Nat. Commun. 2019; 10:4333. 31551417 10.1038/s41467-019-12275-6 PMC6760118

[14] Munson M.J. , O’Driscoll G., Silva A.M., Lázaro-Ibáñez E., Gallud A., Wilson J.T., Collén A., Esbjörner E.K., Sabirsh A A high-throughput Galectin-9 imaging assay for quantifying nanoparticle uptake, endosomal escape and functional RNA delivery. Commun. Biol. 2021; 4:211. 33594247 10.1038/s42003-021-01728-8 PMC7887203

[15] Rhym L.H. , Manan R.S., Koller A., Stephanie G., Anderson D.G. Peptide-encoding mRNA barcodes for the high-throughput in vivo screening of libraries of lipid nanoparticles for mRNA delivery. Nat. Biomed. Eng. 2023; 7:901–910. 37127709 10.1038/s41551-023-01030-4

[16] Thess A. , Grund S., Mui B.L., Hope M.J., Baumhof P., Fotin-Mleczek M., Schlake T. Sequence-engineered mRNA without chemical nucleoside modifications enables an effective protein therapy in large animals. YMTHE. 2015; 23:1456–1464. 10.1038/mt.2015.103 PMC4817881 26050989

[17] Arteta M.Y. , Kjellman T., Bartesaghi S., Wallin S., Wu X., Kvist A.J., Dabkowska A., Székely N., Radulescu A., Bergenholtz J. et al. Successful reprogramming of cellular protein production through mRNA delivered by functionalized lipid nanoparticles. Proc. Natl. Acad. Sci. U.S.A. 2018; 115:E3351–E3360. 29588418 10.1073/pnas.1720542115 PMC5899464

[18] Cheung C.S.F. , Anderson K.W., Wang M., Turko I.V. Natural Flanking Sequences for Peptides Included in a Quantification Concatamer Internal Standard. Anal. Chem. 2014; 87:1097–1102. 25522095 10.1021/ac503697j

[19] Berthold M.R. , Cebron N., Dill F., Gabriel T.R., Kötter T., Meinl T., Ohl P., Thiel K., Wiswedel B. KNIME - the Konstanz information miner: version 2.0 and beyond. ACM SIGKDD Explor. Newslett. 2009; 11:26–31.

[20] Eckardt K.-U. , Kurtz A. Regulation of erythropoietin production. Eur. J. Clin. Invest. 2005; 35:13–19. 10.1111/j.1365-2362.2005.01525.x 16281953

[21] Sedic M. , Senn J.J., Lynn A., Laska M., Smith M., Platz S.J., Bolen J., Hoge S., Bulychev A., Jacquinet E. et al. Safety evaluation of lipid nanoparticle–formulated modified mRNA in the Sprague-Dawley rat and cynomolgus monkey. Vet. Pathol. 2018; 55:341–354. 29191134 10.1177/0300985817738095

[22] Blakney A.K. , Deletic P., McKay P.F., Bouton C.R., Ashford M., Shattock R.J., Sabirsh A. Effect of complexing lipids on cellular uptake and expression of messenger RNA in human skin explants. J. Controlled Release. 2021; 330:1250–1261. 10.1016/j.jconrel.2020.11.033 33250305

[23] Samaridou E. , Heyes J., Lutwyche P. Lipid nanoparticles for nucleic acid delivery: current perspectives. Adv. Drug. Deliv. Rev. 2020; 154–155:37–63. 10.1016/j.addr.2020.06.002 32526452

[24] Evers M.J.W. , Kulkarni J.A., Meel R.v., Cullis P.R., Vader P., Schiffelers R.M State-of-the-art design and rapid-mixing production techniques of lipid nanoparticles for nucleic acid delivery. Small Methods. 2018; 2:1700375.

[25] Alabi C.A. , Love K.T., Sahay G., Yin H., Luly K.M., Langer R., Anderson D.G. Multiparametric approach for the evaluation of lipid nanoparticles for siRNA delivery. Proc. Natl. Acad. Sci. U.S.A. 2013; 110:12881–12886. 23882076 10.1073/pnas.1306529110 PMC3740846

[26] Li J. , Chen Z., Chen F., Xie G., Ling Y., Peng Y., Lin Y., Luo N., Chiang C.-M., Wang H. Targeted mRNA demethylation using an engineered dCas13b-ALKBH5 fusion protein. Nucleic Acids Res. 2020; 48:5684–5694. 32356894 10.1093/nar/gkaa269 PMC7261189

[27] Prima V. , Gore L., Caires A., Boomer T., Yoshinari M., Imaizumi M., Varella-Garcia M., Hunger S.P. Cloning and functional characterization of MEF2D/DAZAP1 and DAZAP1/MEF2D fusion proteins created by a variant t(1;19)(q23;p13.3) in acute lymphoblastic leukemia. Leukemia. 2005; 19:806–813. 15744350 10.1038/sj.leu.2403684

[28] Qin S. , Tang X., Chen Y., Chen K., Fan N., Xiao W., Zheng Q., Li G., Teng Y., Wu M. et al. mRNA-based therapeutics: powerful and versatile tools to combat diseases. Signal Transduct. Targeted Ther. 2022; 7:166. 10.1038/s41392-022-01007-w PMC9123296 35597779

[29] Karikó K. , Muramatsu H., Keller J.M., Weissman D. Increased erythropoiesis in mice injected with submicrogram quantities of pseudouridine-containing mRNA encoding erythropoietin. Mol. Ther. 2012; 20:948–953. 22334017 10.1038/mt.2012.7 PMC3345990

