# Supplementary material for: RNA encoded peptide barcodes enable efficient in vivo screening of RNA delivery systems
Source: Nucleic Acids Res. 2024 Jul 25;52(16):9384–96. doi: 10.1093/nar/gkae648 (PMC11381334; doi:10.1093/nar/gkae648)
Supplement: gkae648_Supplemental_File [file gkae648_supplemental_file.pdf]

Supplementary Information

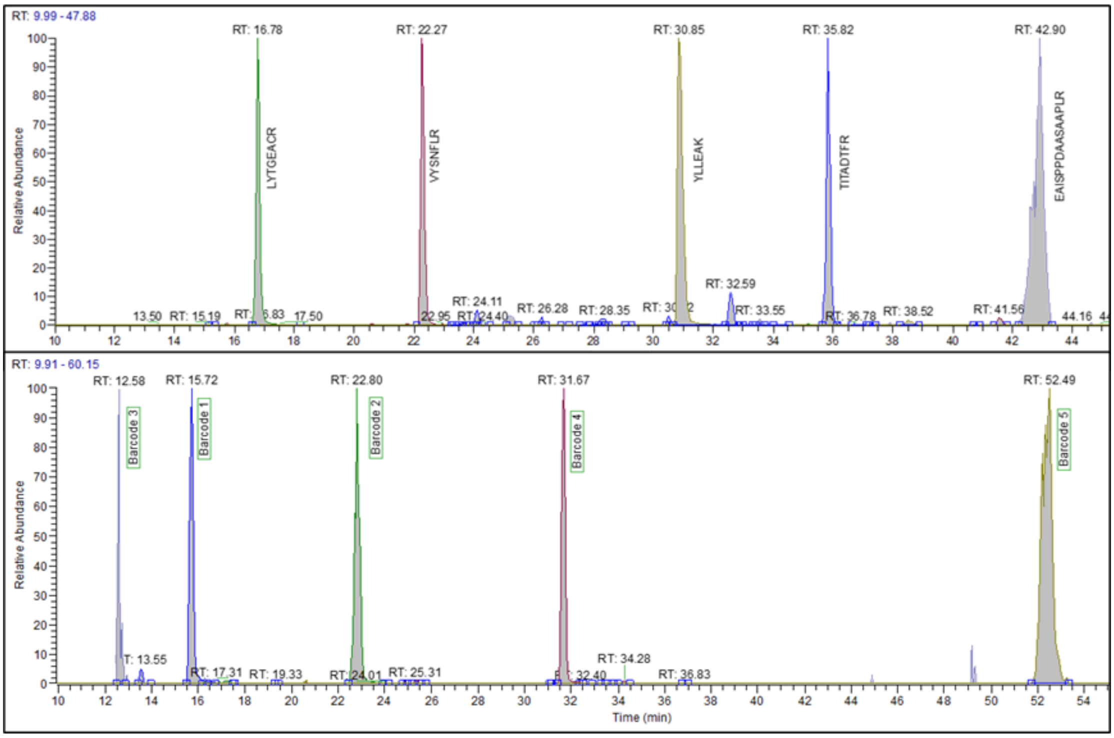

S 1*:* **Barcoded mRNA is fully translated into barcoded hEPO proteins in vivo**. Tandem quantification of EPO protein using representative EPO peptides LYTGEACR (m/z 485.22), TITADTFR (m/z 462.74), YLLEAK (m/z 368.72), VYSNFLR (m/z 449.74) and EAISPPDAASAAPLR (m/z 733.39) as well as five barcoded peptides co-translated from pooled LNPs.

Table S 1: LC-MS/MS method showing accuracy and limits of quantitation

peptide A (fmol/uL)	replicate 1	replicate 2	replicate 3	Average peak area	SD	%CV	experimental C (fmol/uL)	Accuracy	S/N PASS
**1**	3.30E+07	3.35E+07	3.87E+07	3.51E+07	3.18E+06	9.07%	2.11E+00	47%	LLOD
**2**	5.47E+07	5.71E+07	5.96E+07	5.71E+07	2.49E+06	4.36%	2.51E+00	80%	LLOQ
**5**	1.78E+08	1.72E+08	1.74E+08	1.75E+08	3.06E+06	1.75%	4.65E+00	93%	
**10**	4.70E+08	4.14E+08	3.90E+08	4.25E+08	4.08E+07	9.60%	9.19E+00	92%	
**25**	1.40E+09	1.26E+09	1.17E+09	1.28E+09	1.18E+08	9.21%	2.47E+01	99%	
**50**	2.86E+09	2.82E+09	2.59E+09	2.75E+09	1.44E+08	5.24%	5.15E+01	97%	
**75**	4.09E+09	3.87E+09	3.61E+09	3.85E+09	2.38E+08	6.18%	7.15E+01	95%	
**100**	5.51E+09	5.83E+09	5.18E+09	5.51E+09	3.21E+08	5.83%	1.01E+02	99%	
**150**	8.32E+09	8.12E+09	8.15E+09	8.20E+09	1.10E+08	1.34%	1.50E+02	100%	

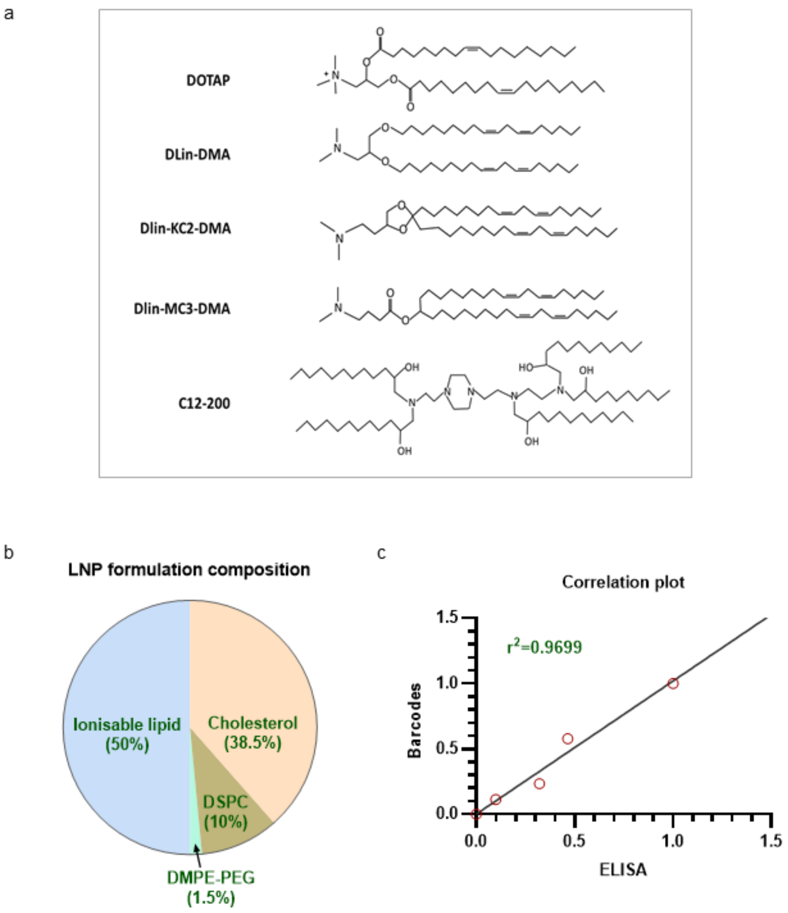

S2: **a.** Structure of lipids used in formulation of LNPs. **b**. Representation of formulation composition of bLNPs used **c.** Correlation plot showing the normalised protein amount measured by PALS and ELISA. Each spot represents the mean normalised amount measured

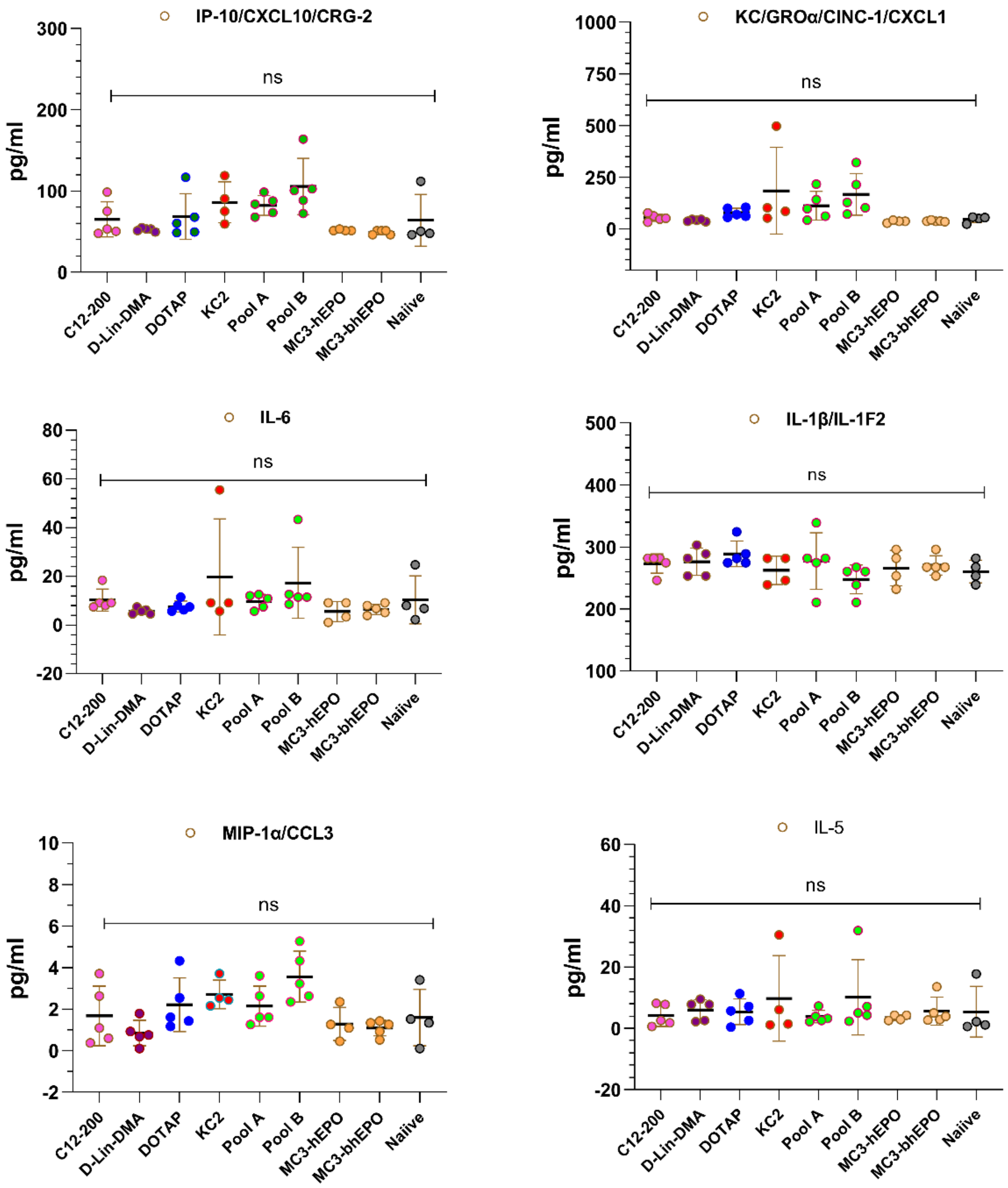

S3: Cytokine assessment of LNP pools in Balb/C mice. Values represent mean ± SD of different cytokines. Statistical significance determined by One-way ANOVA

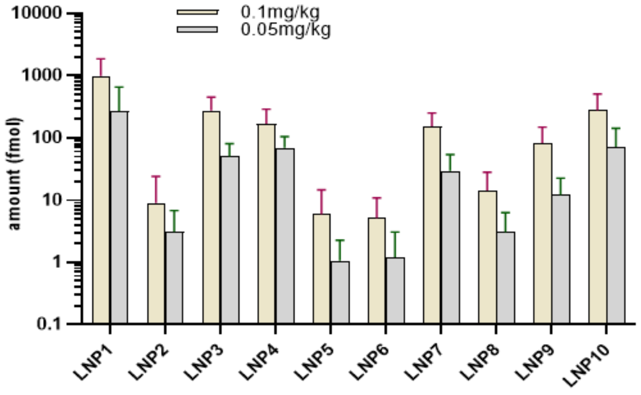

S 4: PALS gives comparable results from different pool sizes. Protein amount measured by PALS from a pool of 10 bLNPs and 5 bLNPs. Bars represent the mean ± SD
